# Correspondence

**Published:** 1954

**Authors:** D. H. Forster


### *Correspondence*

SIR,

We are always hearing about the long time patients have to wait in the Out-Patient Department in Hospitals. Do many of those who sit for long hours on the hard benches really need to be there at all? Was their journey really necessary? I refer to the large number of follow-up cases who will in the end only be seen by a Junior Registrar or perhaps a newly qualified House Surgeon, who will examine their operation scar and satisfy himself that the wound has healed by first intention. Surely the General Practitioner who has first referred the case to the Physician or Surgeon is quite capable of carrying out the follow-up care himself, and referring the patient again to Hospital if he is not satisfied with the progress.

Some of us are becoming tired of the " pinching " of the interesting work which falls to our lot. Is it too late to convince our Consulting Surgeons and Physicians that most General Practitioners are quite capable of doing much of the work which at present clutters up the Out-Patient Departments of our Hospitals? When the G.P. sends a patient up for an opinion he means an *OPINION* as to diagnosis and treatment, and not that the patient shall be taken over permanently by the hospital.

D. H. FORSTER

